# YAP/STAT3 inhibited CD8 ^+^ T cells activity in the breast cancer immune microenvironment by inducing M2 polarization of tumor‐associated macrophages

**DOI:** 10.1002/cam4.6242

**Published:** 2023-06-16

**Authors:** Chen Wang, Ningning Shen, Qingling Guo, Xiaohua Tan, Shaozhong He

**Affiliations:** ^1^ Department of Oncology Ganzhou People's Hospital, The Affiliated Ganzhou Hospital of Nanchang University Ganzhou P. R. China; ^2^ Department of pharmacy Ganzhou Women and Children's Health Care Hospital Ganzhou P. R. China; ^3^ Department of Oncology Binhaiwan Central Hospital of Dongguan Dongguan P. R. China; ^4^ Department of Oncology Shenzhen Third People's Hospital Shenzhen P. R. China

**Keywords:** breast cancer, CD8^+^ T, TAMs, YAP/STAT3

## Abstract

**Background:**

Breast cancer (BC) is the leading cause of cancer‐related death among women. One of the hallmarks of cancer is sustained angiogenesis. YAP/STAT3 may promote angiogenesis and driving BC progression. This study aimed to investigate how YAP/STAT3 affects the immune microenvironment in BC and understand the underlying mechanism.

**Methods:**

To establish a tumor‐associated macrophages (TAMs) model, macrophages were cultured in the 4T1 cell culture medium. A BC mouse model was created by injecting 4T1 cells. The expression of YAP, STAT3, p‐STAT3, VEGF, VEGFR‐2, and PD‐L1 was analyzed using immunofluorescence, western blotting, and quantitative real‐time PCR. Flow cytometry was used to identify M1 and M2 macrophages, CD4^+^ T, CD8^+^ T, and Treg cells. Levels of iNOS, IL‐12, IL‐10, TGF‐β, Arg‐1, and CCL‐22 were measured using enzyme‐linked immunosorbent assay. Co‐IP was used to verify whether YAP binds to STAT3. Hematoxylin–eosin staining was used to observe tumor morphology. Cell counting kit‐8 was selected to detect T‐cell proliferation.

**Results:**

YAP, STAT3, P‐STAT3, VEGF, VEGFR‐2, and PD‐L1 were highly expressed in BC tissues. The M2/M1 macrophages ratio increased in the TAMs group compared with the control group. Inhibiting of YAP and STAT3 decreased the M2/M1 macrophages ratio. YAP was found to bind with STAT3. T‐cell proliferation was enhanced after YAP inhibition, and overexpression of STAT3 reversed the regulation of YAP on T‐cell proliferation. In animal studies, inhibiting YAP inhibited tumor weight and volume development. After YAP inhibition, inflammatory infiltration, M2/M1 macrophage ratio, and Treg cell ratio declined, while CD8^+^ and CD4^+^ T‐cell ratio increased.

**Conclusion:**

In conclusion, this study suggested inhibition of YAP/STAT3 reversed M2 polarization of TAMs and suppressed CD8^+^ T‐cell activity in the BC immune microenvironment. These findings open up new avenues for the development of innovative therapies in the treatment of BC.

## 1 INTRODUCTION

Breast cancer (BC) is the predominant malignant disease afflicting women,^1^ which noticeably contributes to the overall mortality in females across the globe.^2^ It accounts for 23% of all cancer fatalities.^3^ Immunotherapies with immune check point inhibitors are currently considered as one of the most promising strategies for cancer treatment.^4^ However, their clinical application in BC remains restricted.^5^ A large proportion of patients experience not benefit due to tolerance or relapse that may be associated with the presence of multiple immunosuppressive cells within the tumor microenvironment (TME).^6^ The modification of treatment combinations targeting the immunosuppressive TME in BC is crucial for enhancing response to immune checkpoint inhibitors.^7^ Furthermore, the immune landscape of BC highlights susceptibility to immune checkpoint inhibitors.^8^ Thus, this study is aimed at elucidating the mechanism of action of immune checkpoints in BC, which can provide a logical basis for targeted clinical administration of immune checkpoint inhibitors for treating BC.

The signaling pathway involving hippo‐YAP‐associated protein (YAP) is widely discussed as one of the pathways that regulate tumor cell proliferation, migration, and apoptosis,^9^ as well as angiogenesis.^10^ YAP serves as a nuclear transcriptional activator that can trigger the transcription of BC‐related genes.^11^ Furthermore, YAP is also a crucial target in cancer immunity by regulating tumor growth through regulating recruitment and phenotype of TAMs in tumor tissues.^11^, ^12^, ^13^, ^14^ Signal transducer and activator of transcription 3, also known as STAT3, is closely associated with BC progression, proliferation, metastasis, and chemoresistance.^13^ A recent study indicated that YAP/STAT3 promotes the expression of PD‐L1 in M2‐type macrophages of TAMs by activating VEGFR1‐TGF‐β signaling, thus promoting laryngeal cancer immune evasion.^15^ However, the effects of YAP/STAT3 on the immune regulation of BC remain insufficiently understood.

The functions of M1‐type and M2‐type macrophages are almost completely opposite.^16^ Within the TAMs, M1‐type macrophages secrete classical inflammatory cytokines, which induce necrosis in tumor cells.^17^ Conversely, M2‐type macrophages of TAMs have potent tumor‐promoting functions and release immunosuppressive cytokines that impair the function and metabolism of T cells.^18^ The therapeutic effect of immune checkpoint inhibitors on tumor is not solely attributable to CD8^+^ effector T cells but is also noticeably reliant on CD4^+^ helper T cells.^19^ In tumor immunity, CD8^+^ T cells play a pivotal role as tumor suppressors, which cause malignant tumor cell death via physical contact with malignant tumor cells and activating their intracellular signals.^20^ cytotoxic CD8^+^ T cells provide long‐term, effective, and efficient protection to the immune system against intracellular infection and malignant cells.^21^ Therefore, the present investigation aims to assess the effect of the YAP/STAT3 pathway on the BC immune microenvironment.

In summary, this investigation aims to concentrate on the immune checkpoint mechanism in BC while examining study the function of YAP/STAT3 in the immune regulation of BC. Additionally, it aims to analyze the impact of the YAP/STAT3 pathway on the BC immune microenvironment and elucidate its precise mechanism, offering a sound foundation for the treatment of BC using immunosuppressive agents.

## 2 MATERIALS AND METHODS

### 2.1 Patient information

The samples were collected from female patients diagnosed BC recruited from The Second Affiliated Hospital, School of Medicine, The Chinese University of Hong Kong, Shenzhen between June 2020 and July 2022. All patients gave informed consent. Our study selected 6 women with different ages who had been diagnosed with invasive ductal carcinoma. The chosen patients ranged in age from 25 to 65 years old when diagnosed (Table 1). The BC tissue and adjacent paratumor tissues were paired obtained from the BC patients during their initial surgery. Patients who underwent chemotherapy or radiation therapy before surgery were not included. A strictly randomized grouping was utilized to guarantee the statistical significance of our sampling. The study was subjected to accreditation by the IRB of The Second Affiliated Hospital, School of Medicine, The Chinese University of Hong Kong, Shenzhen.

**TABLE 1:** Information of BC patients.

Features	
Age (years)	
<50	3
≥50	3
Menopausal	
Premenopausal	3
Postmenopausal	3
Tumor type	
Luminal B type	4
HER‐2 (HR positive)	2
Tumor size	
≤2.0 cm	3
>2 cm	3
Lymph node metastasis	
No	4
Yes	2
TNM stage	
I	1
II	4
III	1

### 2.2 Primary extraction of spleen single‐cell suspension

Initially, a BALB/c mouse was humanely euthanized via CO_2_. The mice were immediately submerged in 75% ethanol for 5 min. In a sterile environment, a small incision was made in the abdominal region of the mouse, exposing the left side of the abdominal wall. The spleens were then delicately extracted from the mouse using sterile tweezers. The spleen was then placed into cultured blood containing pre‐cooled RPMI‐1640 medium (11875‐119, ThermoFisher Scientific, USA) while snipping off the excess connective tissue and fat. Afterward, the spleen was grinded into the chylous fluid using a frosted slide to gently. Then the liquid was filtered with 70 μm nylon membrane to obtain mouse spleen single‐cell suspension. After that, the cell suspension was centrifuged at a rate of 1500 rpm for a duration of 10 min at 4°C and the supernatant was discarded. Then, 3 mL erythrocyte lysate was added, and the cells were re‐suspended for 10 min. Immediately following this is the addition of 6 mL of complete medium for termination. Subsequently, the liquid was centrifuged at a rate of 1500 rpm for a duration of 10 min, with the supernatant being discarded. Furthermore, the cells were washed twice with PBS (5 times the volume of lysate liquid) at 1500 rpm each time and centrifuged for 10 min. Finally, the cell precipitate was re‐suspended with 1 mL buffer, and cell counts were performed.

### 2.3 Magnetic bead separation of CD8 ^+^ T cells

Magnetic bead separation was performed using mouse CD8^+^ T‐cell isolation kit (130–090‐859, Miltenyi, Germany). Firstly, 5.2 × 10^6^ cells were suspended with 80 μL pre‐cooled buffer. Then 20 μL CD8 magnetic beads were mixed with cells and incubated in the refrigerator for 15 min. Next, 1 mL buffer solution was added. Then the mixture was centrifuge at 1500 rpm for 5 min. After that, the precipitates were collected and re‐suspended with 1 mL buffer. The LS column was placed on the magnetic rack. The column was pre‐balanced with 3 mL buffer. Then treated cells were added to LS column. The column was cleaned with buffer twice. Then 2 mL buffer solution was added to LS column. The cells adsorbed on LS column were collected by piston extrusion for subsequent experiments. Finally, the flow cytometry was used to detect the results of separation of CD8^+^ T cells (Figure S1).

### 2.4 Cell culture and grouping

The cell bank of Chinese Academy of Sciences supplied the 4T1 cell lines (SCSP‐5056) and RAW264.7 mouse macrophages (SCSP‐5036). 4T1 cells and macrophages were grown at 37°C in Dulbecco's modified Eagle's medium (DMEM, d8437‐500 mL, Sigma, USA) with 10% fetal bovine serum (FBS, 10099141, GIBCO, USA). The processing of different groups weres shown in Table S1. The mouse macrophages were randomly divided into 4 different groups: Control, TAMs, TAMs + silence‐negative control (si‐NC), and TAMs + si‐YAP. RAW264.7 cells were cultured normally in the control group. According to previous studies,^22^, ^23^ the RAW264.7 macrophages were co‐cultured with 4T1 cells for 48 h to M2 macrophage polarization in the TAMs group. In the TAMs + si‐NC group, based on the TAMs group, si‐NC was transfected based on the TAMs group. In the TAMs + si‐YAP group, si‐YAP was transfected based on the TAMs group.

To further investigate how YAP affects macrophage polarization through STAT3, the macrophages were further divided into five different groups: TAMs, overexpression negative control (oe‐NC), oe‐YAP, oe‐YAP + si‐NC, and oe‐YAP + si‐STAT3. In the oe‐NC group, based on TAMs group, transfection oe‐NC. In the oe‐YAP group, based on the TAMs group, oe‐YAP was transfected. In the oe‐YAP + si‐NC group, STAT3 was transfected without load based on the oe‐YAP group. In the oe‐YAP + si‐STAT3 group, si‐STAT3 was transfected based on the oe‐YAP group.

To further explore the effect of YAP on T cells, macrophages were divided into six different groups: si‐NC (1:3), si‐NC (1:5), si‐NC (1:10), si‐YAP (1:3), si‐YAP (1:5), and si‐YAP (1:10). In the si‐NC group, based on the si‐NC group, polarized M2 macrophages and CD8^+^ T cells were co‐cultured for 18 h at the ratio of 1:3, 1:5, and 1:10, respectively. In the si‐YAP group, based on the si‐YAP group, polarized M2 macrophages and CD8^+^ T cells were co‐cultured for 18 h at the ratio of 1:3, 1:5, and 1:10, respectively. In the si‐YAP + NC‐oe‐STAT3 and si‐YAP + oe‐STAT3 group, NC‐oe‐STAT3 and oe‐STAT3 were respectively transfected based on the si‐YAP (1:10) group.

### 2.5 Immunofluorescence (IF) staining

First, paraffin section samples of clinical breast cancer tissue and mouse tissue were fixed with 4% paraformaldehyde for 30 min. Then the sections were placed in xylene for 20 min, three times. Next, the sections were placed in 100%, 95%, 85%, and 75% ethanol for 5 min successively. Then the sections were soaked in distilled water for 5 min. The sections were then immersed in EDTA buffer (pH 9.0) and heated to repair the antigens. The sections were then placed in sodium borohydride solution at room temperature for 30 min. Then the sections were soaked in 75% ethanol solution for 1 min. Next, the sections were placed in Sudan black dye solution at room temperature for 15 min. The sections were blocked with 5% bovine serum albumin (BSA) for 60 min. Primary antibody YAP (1:50, 13,584‐1‐AP, PTG, USA), STAT3 (1:50) and p‐STAT3 (1:50) were incubated overnight at 4°C with the sections. The information of antibodies in details were listed in Table 2. Then 50 μL CoraLite488—conjugated affinipure goat anti‐mouse IgG (H + L) (SA00013‐1, Proteintech, USA) or CoraLite594—conjugated goat anti‐rabbit IgG (H + L) (SA00013‐4, 1:100, Proteintech, USA) were added and incubated with sections at 37°C for 90 min. The sections were stained with DAPI (AWC0293a, Abiowell, China) at 37°C for 10 min. Lastly, glycerin was applied to seal the sections, and a fluorescence microscope (BA410T, Motic, Germany) was adopted to analyze the results.

**TABLE 2:** Information of antibodies.

Name	Catalogue number	Source	Dilution rate	Molecular weight (KDa)	Transfer film time (min)	Company
YAP	#14074	Rabbit	1:1000	65–78	98	CST (USA)
STAT3	10253‐1‐AP	Rabbit	1:1000	88	108	Proteintech (USA)
p‐STAT3	ab76315	Rabbit	1:2000	88	108	Abcam (UK)
VEGF	ab32152	Rabbit	1:1000	151	150	Abcam (UK)
VEGFR‐2	26415‐1‐AP	Rabbit	1:500	200	150	Proteintech (USA)
PD‐L1	17952‐1‐AP	Rabbit	1:600	50	70	Proteintech (USA)
β‐Actin	66009‐1‐Ig	Mouse	1:5000	42	60	Proteintech (USA)
HRP goat anti‐mouse IgG	SA00001‐1	Mouse	1:5000	–	90	Proteintech (USA)
HRP goat anti‐rabbit IgG	SA00001‐2	Rabbit	1:6000	–	90	Proteintech (USA)

### 2.6 Western blotting (WB)

RIPA lysate (AWB0136, Abiowell, China) was employed to extract the total protein from tissue and cellular samples of each group, according to the instructions. Protein concentrations were determined utilizing the bicinchoninic acid (BCA) concentration assay kit (ab102536, Abcam, UK). The proteins were separated using SDS gel electrophoresis and then transferred to the nitrocellulose membrane. The membrane was encased in a closed buffer containing 5% skim milk. Subsequently, the membrane and primary antibodies YAP, STAT3, p‐STAT3, VEGF, VEGFR‐2, PD‐L1, and β‐actin were incubated overnight at 4°C. Next, the diluted secondary antibodies HRP goat anti‐mouse IgG and HRP goat anti‐rabbit IgG, were incubated with the membrane at room temperature for 90 min using PBS + 0.05% tween 20 (PBST). Further information regarding antibodies are provided in Table 2. Post incubation, the membrane was treated with ECL chemiluminescent solution (AWB0005, Abiowell, China) for 1 min. Finally, the membrane was analyzed in a chemiluminescent imaging system (Chemiscope6100, CLiNX, China). β‐actin was used as an internal reference protein.

### 2.7 Real‐time quantitative PCR (RT‐qPCR)

Following the manufacturer's instructions, total RNA samples were extracted from BC cells and tissues using the Trizon reagent (15596–026, Thermo Fisher Scientific, USA). The mRNA was reverse transcribed to cDNA with the mRNA reverse transcription kit (CW2569, CWBIO, China). Primers were designed by primer 5 software and the results are shown in Table 3. β‐actin served as an internal reference gene. The experiment was performed on fluorescence quantitative PCR instrument (PIKOREAL96, Thermo Fisher, USA).

**TABLE 3:** Sequences of the primers.

Gene name	Forward (5′–3′)	Reverse (5′–3′)
YAP	ATGGAGGGACTCCGAATG	GGTGCCACTGTTAAGAAAGG
VEGF	GAACCAGACCTCTCACCGGAA	ACCCAAAGTGCTCCTCGAAG
VEGFR‐2	ACTGTGGCGAAGATGTTTTTGAGC	CGGACTTGACTGCCCACT
β‐actin	ACATCCGTAAAGACCTCTATGCC	TACTCCTGCTTGCTGATCCAC

### 2.8 Flow cytometry

The cell suspension was taken 100 μL cells to a 1.5 mL centrifuge at a concentration of 1 × 106 mL‐1. Each tube was added with monoclonal antibodies specifically binding to different macrophage and lymphocyte subsets according to the detection requirements and incubated for 30 min at room temperature in the dark. Finally, the proportion of cells was collected and determined by flow cytometry (A00‐1‐1102, Beckman, USA). The following monoclonal antibodies were employed in the experiment: F4/80 (BM8, 11‐4801‐82), CD16/CD32 (93, 17‐0161‐82), CD206 (685641, MA5‐23594), CD3e (145‐2C11, MA5‐17658), CD4 (GK1.5, 12‐0041‐82), CD8a (53‐6.7, 17‐0081‐82), CD25 (PC61.5, 17‐0081‐82), and FOXP3 (FJK‐16s, 17‐5773‐82), which all obtained from the USA Thermo Fisher Scientific Co. Ltd.

### 2.9 Enzyme‐linked immunosorbent assay (ELISA)

The centrifuged macrophages and serum were quantitatively analyzed according to the operating instructions of the kit. The kit of iNOS (CSB‐E08326m, CUSABIO, China), IL‐12 (CSB‐E04600m, CUSABIO, China), IL‐10 (CSB‐E04594m, CUSABIO, China), TGF‐β1 (CSB‐E04726m, CUSABIO, China), Arg1 (CSB‐EL002005MO, CUSABIO, China), and CCL‐22 (CSB‐E04661m, CUSABIO, China) were used to examine the content of iNOS, IL‐12, IL‐10, TGF‐β1, Arg1, and CCL‐22 in macrophages and serum of mice in each group, respectively.

### 2.10 Co‐immunoprecipitation (Co‐IP)

The 0.030 g tissue were digested with 500 μL IP lysate (AWB0144a, Abiowell, China) in a biological sample homogenizer (BioPrep‐24, ALLSHENG, China). The treated samples were placed on ice for cracking 30 min. The samples were then centrifuged at 4°C at 12000 rpm for 15 min. Then the centrifuged supernatant was transferred to a 1.5 mL centrifuge tube. The protein supernatants were divided into three groups: Input, IgG, and IP group (YAP or STAT3). In the IgG group, 1.5 mL normal rabbit IgG (2 μg, B900610, Proteintech, USA) was added. No antibodies were added in the Input group. IP group was added with 2 μL of target antibodies (YAP or STAT3) and incubated overnight at 4°C. Then 20 μL Protein A/G agarose beads were mixed with 200 μL IP cracking solution. Then the mixture was centrifuged at 3000 rpm for 3 min. This step repeated four times to obtain the precipitation. The cell lysate incubated with the antibody overnight was added to the pre‐treated Protein A/G agarose beads, which were slowly shaken at 4°C for 2 h to conjugate the antibody to Protein A/G agarose. After Co‐IP, the mixtures were centrifuged at 3000 rpm at 4°C for 3 min. After centrifugation, agarose beads were placed at the bottom of the tube and supernatant was discarded. The agarose beads were washed 4 times with 400 μL IP cracking solution and the precipitation was collected. Next, the collected agarose bead precipitate was mixed with 30 uL IP lysate and 10 uL 5 × loading buffer (15 uL 5 × loading buffer was added in the Input group). The mixtures were boiled with boiling water for 5 min. Then them were cooled in an ice box rapidly. After that, the treated samples were used for WB experiment. The procedures of WB assay in detail were listed in Section 2.6. The primary antibodies used in this study were STAT3 and YAP. The secondary antibodies were HRP goat anti‐mouse IgG and HRP goat anti‐rabbit IgG. Specific antibody information are shown in Table 2

### 2.11 Cell counting kit‐8 (CCK‐8)

The co‐cultured macrophages and CD8^+^ T cells were seeded in 96‐well plates at a density of 1 × 10^4^ cells/well. CCK‐8 (NU679, DOJINDO, Japan) was used for examination. Incubation was performed at the condition of 37°C containing 5% CO_2_ for 4 h. The optical density (OD) value at 450 nm was measured utilizing the Microplate reader (MB‐530, HEALCES, China).

### 2.12 Lactate dehydrogenase (LDH) release method

Macrophages with different pretreatment were co‐cultured with CD8^+^ T cells for 18 h. The supernatants were collected and detected following the instructions of the LDH detection kit (A020‐2, Nanjing Jiancheng Bioengineering Research Institute, China). The OD value at 450 nm was measured using a microplate reader.

### 2.13 Animal experiment

Hunan SJA Laboratory Animal Co., Ltd supplied 4‐week‐old BALB/c mice for our research. The experiments on animals were reviewed and approved by the Experimental Animal Ethic Committee of The Second Affiliated Hospital, School of Medicine, The Chinese University of Hong Kong, Shenzhen (2022054DW). The Guide for the Care and Use of Laboratory Animals was followed in our care of the animals. After 1 week of adaptive feeding, the mice were separated into three different groups: model, sh‐NC, and sh‐YAP. In the model group, we injected 1 × 10^6^ 4T1 cells subcutaneously into the chest wall of BALB/c mice between intercostals 5 and 6. One week after the tumor was seeded, the mice were randomly divided into groups according to the size of the tumor and entered the stage of drug intervention. In the sh‐NC group, each mouse was injected with 50 μL YAP interfering adenovirus no‐load (1 × 10^8^ pfu, NM_001130145, HANBIO, China) in the tail vein. In the sh‐YAP group, 50 μL YAP interfering adenovirus (1 × 10^8^ pfu) was injected into each mouse tail vein.^24^ A 2‐week drug intervention was conducted once a week. After the last dose, blood was taken, and the mice were euthanized. The tumor was carefully dissected and eliminated. The tumor was positioned neatly and photographed. The tumor volume and weight were measured with a ruler and scale.

### 2.14 Hematoxylin–eosin (HE)‐staining

The tumor tissues were washed with cold saline. Then were dried with filter paper. After the tissues were fixed for 24 h with 4% paraformaldehyde, they were embedded in paraffin. Sections were dewaxed with xylene and hydrated with gradient alcohol after baking at 60°C for 1 h. Hematoxylin and eosin were stained sequentially on each section. Then we sealed the dehydrated sections with neutral gum and observed them under a light microscope.

### 2.15 Statistical analysis

GraphPad prism 9 was determined as the main application software for the analysis and statistics of experimental data, and mean ± standard deviation was used to express the data. The *t*‐test, one‐way analysis of variance (ANOVA), and two‐way ANOVA were adopted for group comparisons. *p* < 0.05 indicated that the difference between groups is statistically significant.

## 3 RESULTS

### 3.1 YAP and STAT3 were highly expressed in BC tissues

**FIGURE 1:**
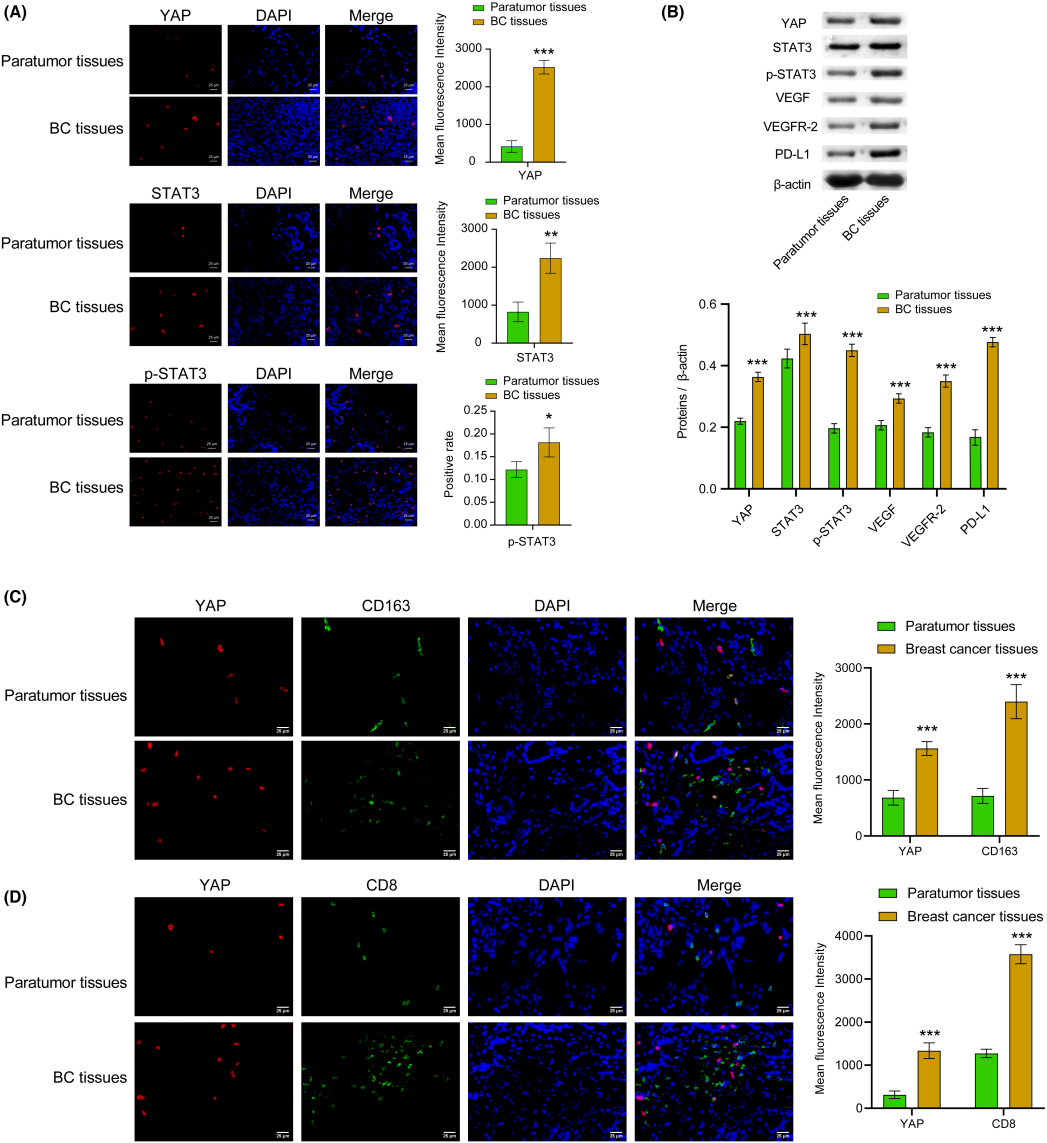
YAP and STAT3 were highly expressed in the BC tissues. (A) IF staining was utilized to identify the YAP, STAT3, and p‐STAT3 expression in tissues. The red signal represented positive staining for YAP, p‐STAT3, and STAT3, while the blue signal indicated nuclear staining. (B). WB was selected to examine the levels of the YAP, STAT3, p‐STAT3, VEGF, VEGFR‐2, and PD‐L1 proteins in tissues. (C) The co‐localization of YAP and M2 macrophage (CD163) in tissues were detected using IF. (D) The co‐localization of YAP and CD8 (CD68/CD163) in tissues was analyzed using double IF. The positive signal was CD163 (green) + YAP (red), CD8 (green) + YAP (red). The blue was nuclear staining signal. All data were presented as mean ± SD (*n* = 3) from three independent experiments, each performed in triplicate. ***p < 0.001, **p < 0.01, *p < 0.05 vs the paratumor tissues group. *t*‐test.

To investigate the expression of YAP and STAT3 in BC tissues, we collected both BC tissues and adjacent paratumor tissue samples from six patients with BC. Positive expression of YAP, STAT3, and p‐STAT3 was represented in red. We observed a significant increase in the expressions of YAP, STAT3, and p‐STAT3 in BC tissues compared to paratumor tissue. Moreover, WB analysis revealed higher levels of YAP, STAT3, p‐STAT3, VEGF, VEGFR‐2, and PD‐L1 in the BC tissues and paratumor tissue. The findings suggested a marked elevation of YAP, STAT3, p‐STAT3, VEGF, VEGFR‐2, and PD‐L1 expression levels in the BC tissues (Figure 1A,B). Compared with the paratumor tissue, the co‐localization of YAP with M2 macrophages noticeably increased in BC tissue (Figure 1C). Moreover, the co‐localization of YAP and CD8 was markedly higher in the BC tissue than in the paratumor tissue (Figure 1D). These results indicated that YAP and STAT3 highly expressed in BC tissues. Indeed, increased YAP levels might be associated with CD8^+^ T cell infiltration, though further investigation is needed.

### 3.2 Inhibition of YAP reversed macrophages M2‐type polarization induced by the supernatant of BC cells

**FIGURE 2:**
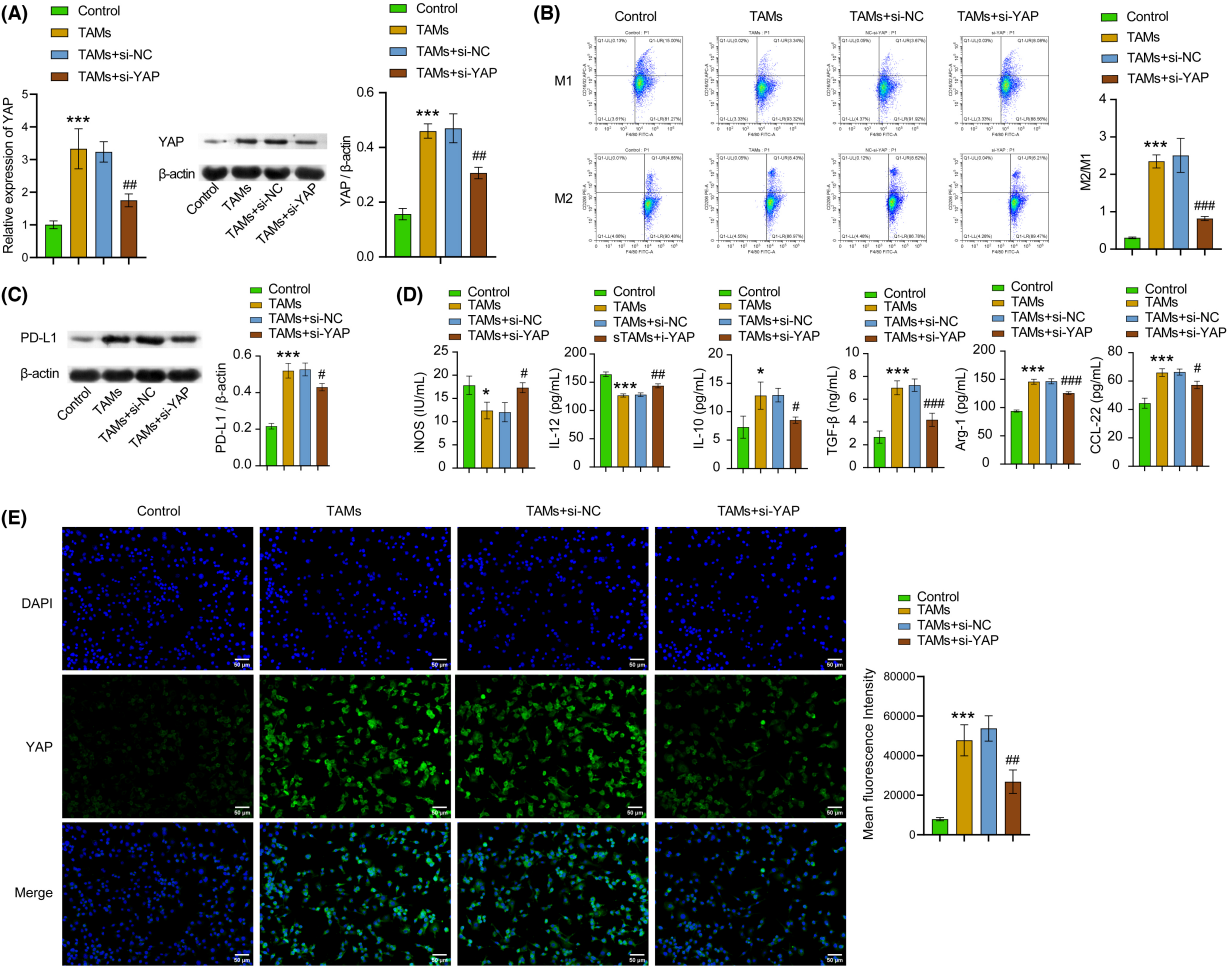
Inhibition of YAP reversed M2 macrophage polarization induced by the supernatant of BC cells. (A) RT‐qPCR and WB were chosen to detect YAP expression in cell lines. (B) M1 and M2 macrophages were identified with flow cytometry with markers of CD16/32^+^F4/80^+^ and CD206^+^F4/80^+^, respectively. (C) The expression of PD‐L1 protein in cell lines was detected through WB. (D) The iNOS, IL‐12, IL‐10, TGF‐β, Arg‐1, and CCL‐22 expressions in cell lines were tested with ELISA. (E) The expression of YAP in cell lines was examined with IF staining. The green signal indicated positive staining for YAP, while the blue signal indicated nuclear staining. All data were presented as mean ± SD (*n* = 3) from three independent experiments, each performed in triplicate. ***p < 0.001, **p < 0.01, *p < 0.05 vs the control group, ^###^ *p* < 0.001, ^##^ *p* < 0.01, ^#^ *p* < 0.05 vs the TAMs + si‐NC group. one‐way ANOVA (A–E).

Subsequently, we investigated the impact of YAP on macrophage M2‐type polarization induced by the supernatant of BC cells. To establish an in vitro TAMs model, we cultured macrophages in 4T1 cell culture medium. First, we performed the transfection efficiency verification experiment. Our results suggested the transfection of si‐YAP noticeably decreased the expression of YAP in mRNA and proteins levels. The transfection efficiency was exceeding 70%, indicating a successful transfection (Figure S2). Following this, we detected the levels of YAP and PD‐L1 and changes in macrophage phenotype after silencing YAP. Results demonstrated that silencing YAP reversed the elevation of YAP and PD‐L1 expression levels, the ratio of M2/M1 macrophages, and contents of M2‐type macrophage markers (IL‐10, TGF‐β, ARG‐1, and CCL‐22) in the TAMs group. In addition, there was a decrease in the content of M1‐type markers (iNOS and IL‐12) in the TAMs + YAP group compared to the TAMs + si‐NC group (Figure 2A–E, Figure S3). The experimental findings support the conclusion that inhibition of YAP impedes M2 macrophage polarization. Overall, YAP played a pivotal role in regulation macrophage M2‐type polarization.

### 3.3 YAP/STAT3 regulated macrophages M2‐type polarization induced by BC cell supernatant

**FIGURE 3:**
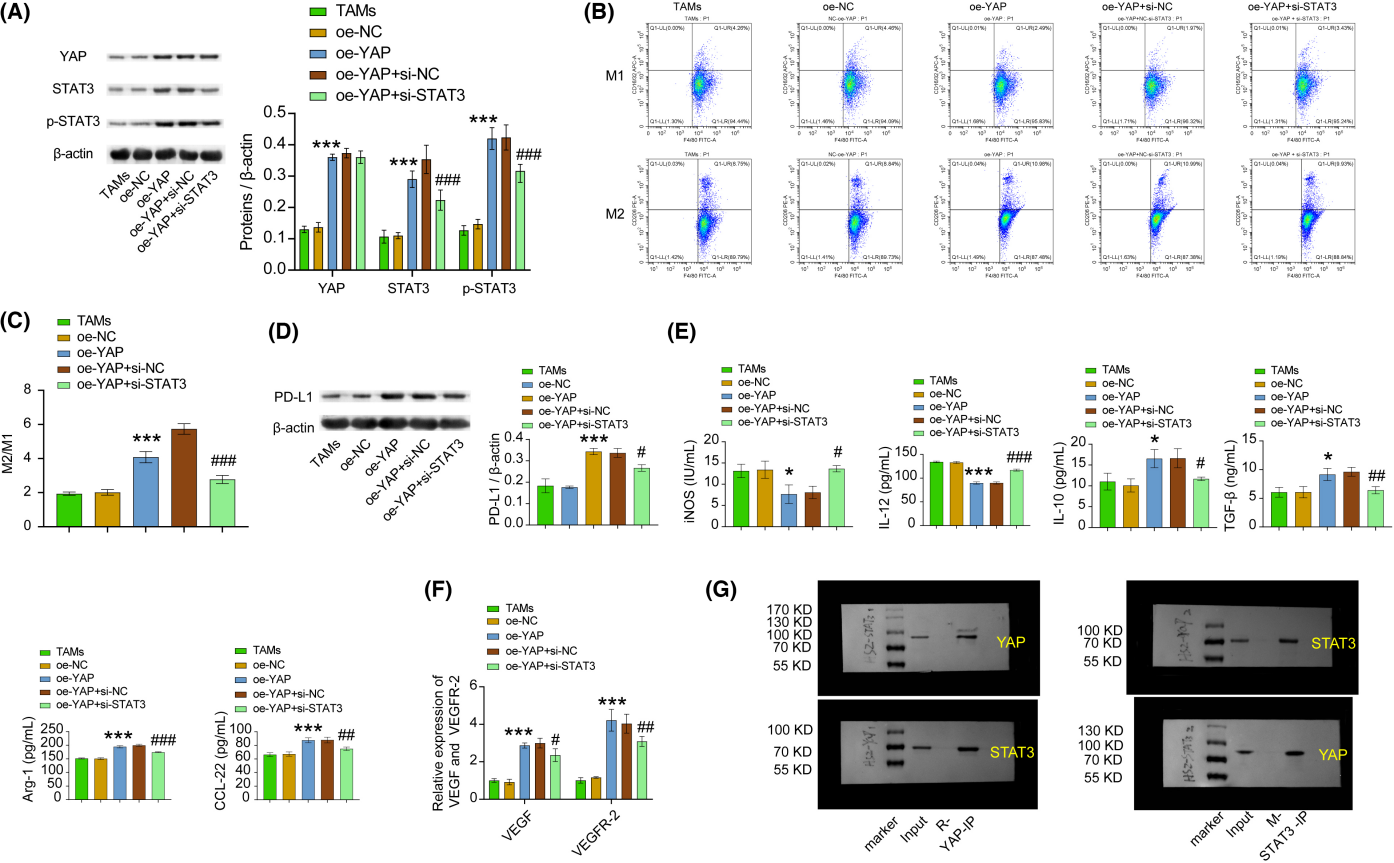
YAP/STAT3 regulated M2 macrophage polarization induced by BC cell supernatant. (A) YAP, STAT3, and the p‐STAT3 expression level in cell lines were detected by WB. (B and C) M1 and M2 macrophages were identified using flow cytometry. (D) PD‐L1 expression level in cell lines was detected by WB. (E) ELISA was adopted to test iNOS, IL‐12, IL‐10, TGF‐β, Arg‐1, and CCL‐22 expression in cell lines. (F) RT‐qPCR was used to detect the expression of VEGF and VEGFR‐2 in cell lines. (G) CoIP verified that YAP directly binds to STAT3 protein in cell lines. ***p < 0.001, **p < 0.01, *p < 0.05 vs the oe‐NC group, ^###^ *p* < 0.001, ^##^ *p* < 0.01, ^#^ *p* < 0.05 vs the oe‐YAP + si‐NC group. All data were presented as mean ± SD (*n* = 3) from three independent experiments, each performed in triplicate. one‐way ANOVA.

The expression of VEGF and VEGFR2 can be regulated by M2 macrophages, thereby promoting the development of BC.^25^ In this study, we examined the influence of YAP and STAT3/VEGF/VEGFR2 pathways on macrophages M2‐type polarization induced by BC cell supernatant. Based on the in vitro TAMs model construction, we observed changes in the expression levels of YAP, STAT3, P‐STAT3, PD‐L1, VEGF, and VEGFR‐2, as well as macrophage phenotype, upon overexpressing YAP. Compared with the oe‐NC group, the oe‐YAP group exhibited dramatically higher expression levels of YAP, STAT3, p‐STAT3, PD‐L1, VEGF, and VEGFR‐2, as well as higher ratio of M2/M1 macrophages, and increased expression of IL‐10, TGF‐β, Arg‐1, and CCL‐22. Conversely, expression levels of iNOS and IL‐12 were markedly reduced. In contrast to the oe‐YAP + si‐NC group, the oe‐YAP + si‐STAT3 group showed remarkably decreased expression levels of STAT3, p‐STAT3, PD‐L1, VEGF, and VEGFR‐2, as well as a lower ratio of M2/M1 macrophages, and reduced expression of IL‐10, TGF‐β, Arg‐1, and CCL‐22. However, expression levels of iNOS, IL‐12 were dramatically increased (Figure 3A–F). CoIP assay results demonstrated the interaction between YAP and STAT3 protein (Figure 3G). Our results suggested that YAP interacted with STAT3 to regulate VEGF and VEGFR‐2 expression, ultimately promoting macrophage M2‐type polarization.

### 3.4 YAP/STAT3 promoted M2‐type polarization of TAMs and inhabited CD8 ^+^ T cell viability

**FIGURE 4:**
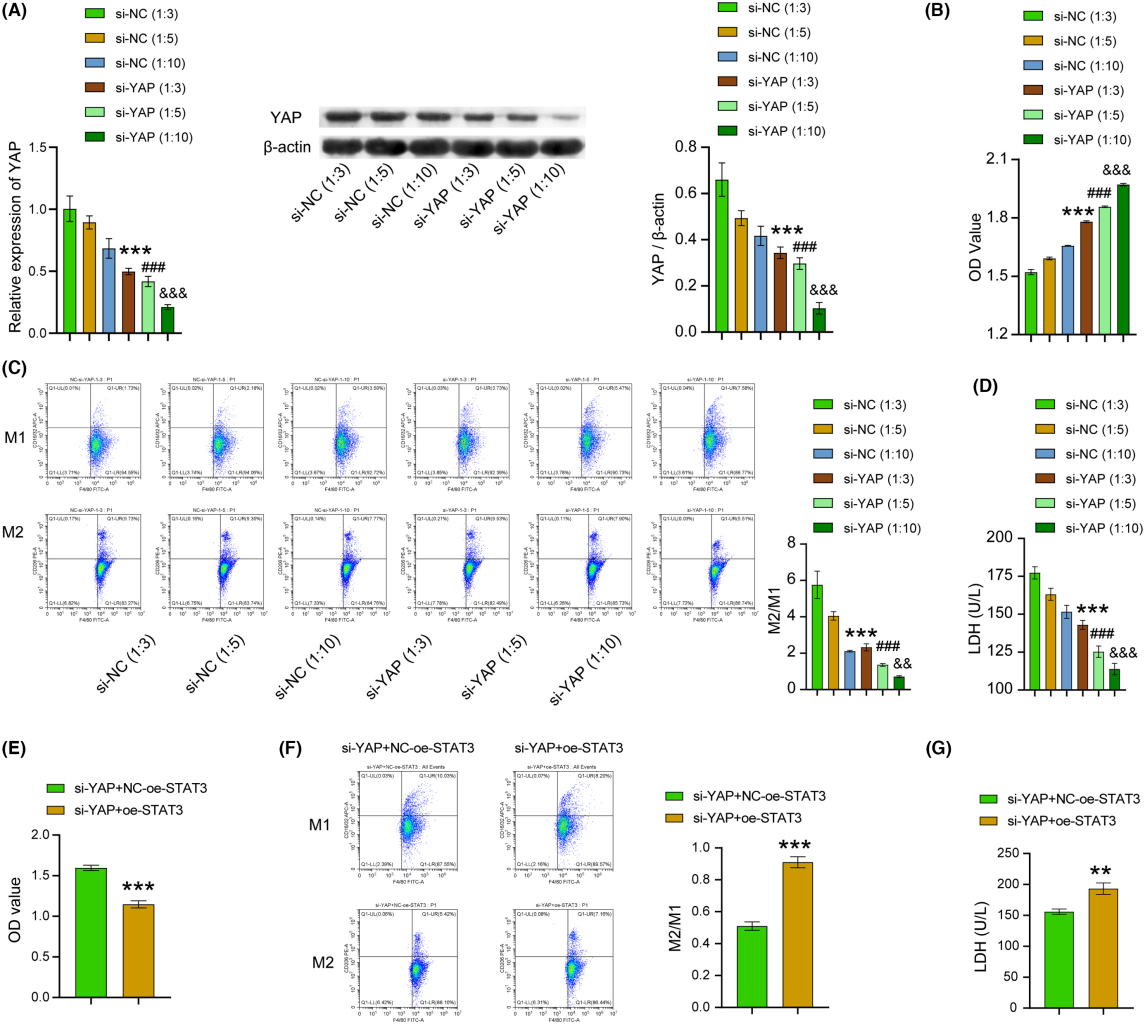
YAP/STAT3 promoted M2 polarization of TAMs and inhabited CD8^+^ T cell viability. (A) WB and RT‐PCR were chosen to test the expression of YAP in cell lines. (B) CCK8 was selected to examine the proliferation of T cells in cell lines. (C) M1‐ and M2‐type macrophages in cell lines were identified using flow cytometry. (D) T‐cell‐mediated cytotoxicity in cell lines was investigated by the LDH release assay. ***p < 0.001, **p < 0.01, *p < 0.05 vs the si‐NC (1:3) group, ^###^ *p* < 0.001, ^##^ *p* < 0.01, ^#^ *p* < 0.05 vs the si‐NC (1:5) group, ^&&&^ *p* < 0.001, ^&&^ *p* < 0.01, ^&^ *p* < 0.05 vs the si‐NC (1:10) group. One‐way ANOVA. (E) CCK8 was selected to examine the proliferation of T cells in cell lines. (F) M1‐ and M2‐type macrophages in cell lines were identified using flow cytometry. (G) T cell‐mediated cytotoxicity in cell lines was investigated by the LDH release assay. ***p < 0.001, **p < 0.01, *p < 0.05 vs the si‐YAP + NC‐oe‐STAT3. *t*‐test. All data were presented as mean ± SD (*n* = 3) from three independent experiments, each performed in triplicate.

To explore the effect of YAP on TAMs, we assessed the YAP expression, CD8^+^ T cell proliferation, and defined macrophage phenotype using an in vitro TAMs model. Our findings revealed a significant reduction in YAP expression level, as well as in the proportion of M2/M1 macrophages and LDH content in the si‐YAP group when compared to the si‐NC group. Additionally, T‐cell proliferation remarkably enhanced and showed a concentration‐dependent alteration (Figure 4A–D). Furthermore, compared with the si‐YAP + NC‐oe‐STAT3 group, the si‐YAP + oe‐STAT3 group displayed a substantial decrease in T‐cell proliferation and a significant increase in the proportion of M2/M1 macrophages and LDH content (Figure 4E–G). As the results, inhibiting YAP expression inhibited macrophage M2‐type polarization and promoted CD8^+^ T cell viability. Consequently, these results indicated that YAP/STAT3 promoted M2 polarization of TAMs and inhabited CD8^+^ T‐cell viability.

### 3.5 Inhibition of YAP could affect TME and tumor proliferation through the STAT3/VEGF/VEGFR‐2 axis

**FIGURE 5:**
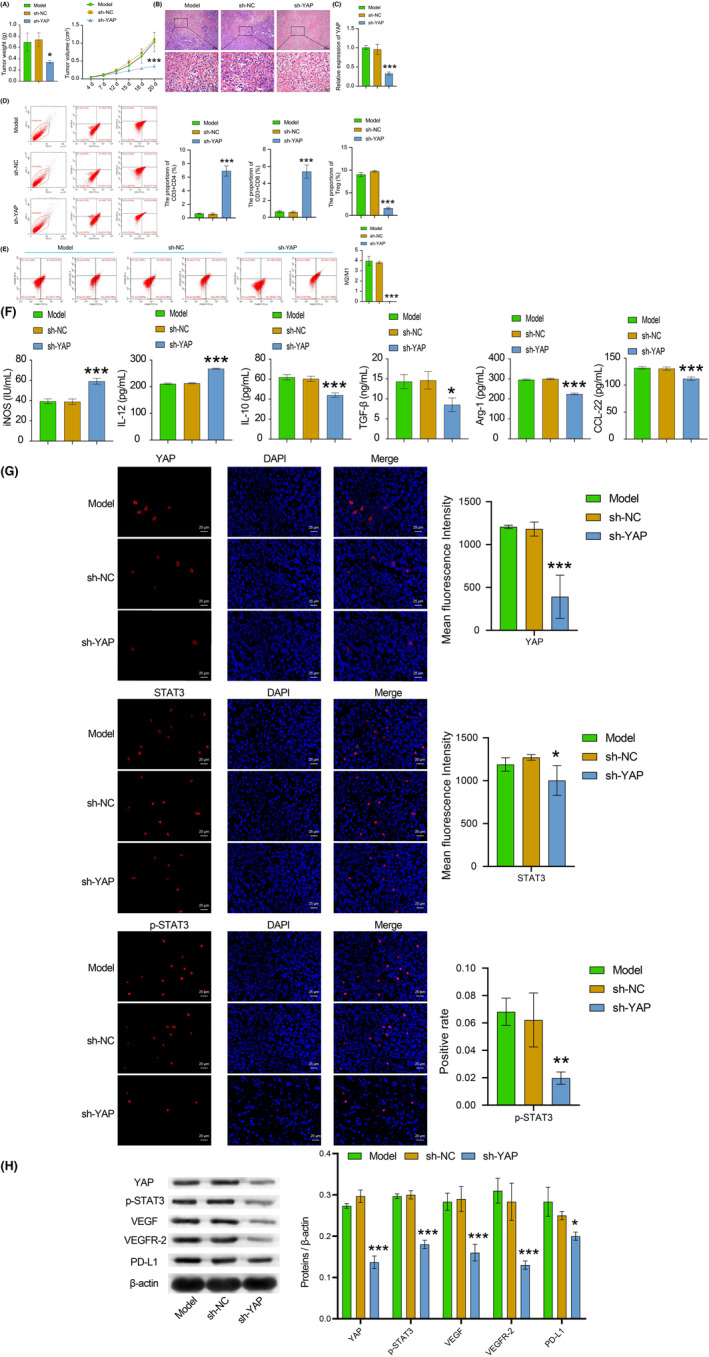
Inhibition of YAP could affect TME and tumor proliferation through the STAT3/VEGF/VEGFR‐2 axis. (A) The detection of volume and weight of tumors of mice. (B) The tumor morphological changes of mice were observed by HE staining. (C) YAP expression of tumor tissues was tested by RT‐qPCR. (D) CD3^+^CD4^+^, CD3^+^CD8^+^, and treg cells in tumor tissues were examined by flow cytometry. (E) The ratio of M1 and M2 macrophages in tumor tissues was tested by flow cytometry. (F) The iNOS, IL‐12, IL‐10, TGF‐β, Arg‐1, and CCL‐22 expressions were tested by ELISA. (G) IF staining was selected to examine the YAP, STAT3, and p‐STAT3 expression levels in tumor tissues. The red signal represented positive staining for YAP, p‐STAT3, and STAT3, while the blue signal indicated nuclear staining. (H) The levels of YAP, STAT3, p‐STAT3, VEGF, VEGFR‐2, and PD‐L1 in tumor tissues were examined by WB. All data were presented as mean ± SD (*n* = 3) from three independent experiments, each performed in triplicate. ***p < 0.001, **p < 0.01, *p < 0.05 vs the model group. One‐way ANOVA (A, left and B–H). Two‐way ANOVA (A, right).

Then, we proceeded to investigate the impact of YAP on TAMs. To further investigate, we established a BC mice model using BALB/c mice and examined the expression of YAP and proliferation of CD8^+^ T cells, as well as the changes in M1 and M2 macrophage phenotype. We found that compared with the sh‐NC group, the sh‐YAP group showed dramatical decreased in tumor weight and volume, number of tumor cells, YAP protein expression, Treg cells, M2/M1 macrophages ratio, IL‐10, TGF‐β, Arg‐1, and CCL‐22 expressions, as well as YAP, STAT3, p‐STAT3, PD‐L1, VEGF VEGFR‐2 expressions. The sh‐YAP group showed noticeable increase in the numbers of CD4^+^ T cells and CD8^+^ T cells, iNOS and IL‐12 expressions when compared to the sh‐NC group (Figure 5A–H). Our study revealed that inhibiting YAP expression could hinder tumor growth and macrophages M2‐type polarization, activate Treg cells, activate CD4^+^ and CD8^+^ T cells, and regulate the expression of YAP, STAT3, p‐STAT3, PD‐L1, VEGF, and VEGFR‐2. As a result, our experiments indicated that inhibition of YAP could affect TME and tumor proliferation at least through the STAT3/VEGF/VEGFR‐2 axis.

## 4 DISCUSSION

STAT3 has been found to interact with YAP in the acceleration of BC development.^26^ Overexpression of YAP in BC cells is more likely to proliferate, transform, migrate, and invade, as well as other processes that drive tumorigenesis and metastasis.^27^ Extant research denotes that YAP interacts with STAT3 to promote endothelial cell proliferation, migration, and the formation of tumor angiogenesis via VEGF.^28^, ^29^ As evidenced by experimental results, the STAT3/VEGFR2 axis can promote tumor development.^30^ Therefore, it is apparent that YAP/STAT3/VEGF/VEGFR2, and other pathways are closely function in the regulating of the occurrence and development of cancer, while there are few studies on this pathway in BC. In this study, through expression analysis of related proteins in BC and paratumor tissue samples, we confirmed high levels of expression of YAP, STAT3, p‐STAT3, VEGF, VEGFR‐2, and PD‐L1 in BC tissues.

According to reports, up‐regulation of YAP in M2‐type macrophages has been associated with the development of triple‐negative BC.^31^ Activation of the STAT3 signaling pathway may lead to macrophage M2‐type polarization and PD‐L1 expression.^32^ Despite this, the specific mechanism of YAP and STAT3 interaction in BC still unknown. In this study, a TAMs model was constructed by inducing macrophage RAW264.7 with 4T1 cell medium. Furthermore, a BC mouse model was established by injecting 4T1 cells into the mammary gland of female BALB/ C mice. The results showed that macrophages M2‐type polarized along with YAP and PD‐L1 expression increased of during BC development. After inhibition of YAP gene, macrophage M2‐type polarization was inhibited along with the decrease levels of YAP and PD‐L. Overexpression of the YAP gene had the opposite effect. After overexpression of the YAP gene, YAP, STAT3, P‐STAT3, PD‐L1, VEGF, and VEGFR‐2 expression levels were markedly increased, and macrophages were M2‐type polarized. After silencing STAT3 protein in the oe‐YAP group, YAP, STAT3, P‐STAT3, PD‐L1, VEGF, and the VEGFR‐2 expression level were dramatically decreased, and macrophage M2‐type polarization was inhibited. The Co‐IP assay confirmed the interaction between YAP and STAT3 protein. VEGF can up‐regulate the level of VEGFR2, thereby activating the phosphorylation of the JAK2‐STAT3 pathway and promoting tumor angiogenesis.^33^ Inhibiting VEGFR2/STAT3/HIF‐1α axis signaling pathway can inhibit the proliferation, migration, and tumor angiogenesis of hucCT‐1 and RBE cells.^34^ Additionally, this study demonstrated that YAP/STAT3 signaling axis could promote tumor angiogenesis. In conclusion, YAP proteins interacted with STAT3 pathway proteins to promote the macrophage M2‐type polarization induced by BC cell supernatant. These findings shed new light on the mechanism of YAP and STAT3 regulation of BC.

YAP was reported to promote up‐regulation of PD‐L1 in BRAFI‐resistant melanoma cells, facilitating evasion of immune responses mediated by cytotoxic T cell.^35^ Similarly, in laryngeal carcinoma, YAP/STAT3 was found to promote immune evasion by activating VEGFR1‐TGFβ signaling and enhancing PD‐L1 expression in the M2‐type macrophage of TAMs.^15^ Therefore, in the present study, the immune regulation of YAP/STAT3 in BC was further investigated. The findings suggested that YAP gene inhibition led to macrophage M2‐type polarization and remarkably enhanced of T‐cell proliferation. After inhibition of the YAP, macrophage M2‐type polarization was further inhibited with the increase of the proportion of co‐cultured T cells. Furthermore, oe‐STAT3 reversed the regulation of si‐YAP. Remarkably, animal experiments showed similar trends, as reduction in tumor weight and volume, as well as tumor cell numbers, inhibition of CD4^+^ and CD8^+^ T cells, and macrophage M2‐type polarization upon silencing YAP. All in all, these results indicated that YAP/STAT3 facilitated M2‐type polarization of TAMs and inhibited immune activity of T cells.

## 5 CONCLUSIONS

This research demonstrated that YAP/STAT3 inhibited the activity of CD8^+^ T cells in the immune microenvironment of BC via promoting M2‐type polarization of TAMs. This established a reliable theoretical basis for the development of anti‐BC drugs. We will plan to employ the DuoLink kit to confirm the interaction between YAP1 and STAT3 in tissues. We will also design further experiments to evaluate the potential of modulating TAMs or tumor cells by interfering with YAP to counteract the TME. Going forward, we plan to collect fresh samples with differing YAP expression levels for flow cytometry analysis, in order to gain a better understanding of the correlation between YAP expression and T‐cell infiltration. Moreover, in future studies, drug studies targeting YAP/STAT3 may be conducted to provide invaluable theoretical support for the treatment of BC.

## AUTHOR CONTRIBUTIONS

**Chen Wang:** Formal analysis (equal); visualization (equal). **Ningning Shen:** Writing – review and editing (equal). **Qingling Guo:** Conceptualization (equal); data curation (equal); formal analysis (equal); visualization (equal); writing – original draft (equal). **Xiaohua Tan:** Writing – review and editing (equal). **Shaozhong He:** Conceptualization (equal); data curation (equal); formal analysis (equal); funding acquisition (equal); visualization (equal); writing – original draft (equal).

## FUNDING INFORMATION

This work was supported by the National Natural Science Foundation of China (No. 81860468, No. 81660435), Natural Science Foundation of Guangxi (No. 2018GXNSFAA281324), Shenzhen High–level Hospital Construction Fund (No. G2022091), and The Special Funds for Strategic Emerging Industry of Shenzhen (No. F‐2022‐Z99‐502266).

## CONFLICT OF INTEREST STATEMENT

The authors declare that there is no conflict of interest regarding the publication of this paper.

## ETHICS STATEMENT

The study was subjected to accreditation by the IRB of The Second Affiliated Hospital, School of Medicine, The Chinese University of Hong Kong, Shenzhen. All experiments conformed to the ethical standards of the Declaration of Helsinki. The experiments on animals were reviewed and approved by the Experimental Animal Ethic Committee of The Second Affiliated Hospital, School of Medicine, The Chinese University of Hong Kong, Shenzhen. The Guide for the Care and Use of Laboratory Animals was followed in our care of the animals.

## Supporting information


Table S1.

Figure S1.

Figure S2.

Figure S3.

Figures S1–S3 Captions

## Data Availability

The data used to support the findings of this study are included within the article.
