# Supplementary material for: YAP/STAT3 inhibited CD8 + T cells activity in the breast cancer immune microenvironment by inducing M2 polarization of tumor‐associated macrophages
Source: Cancer Med. 2023 Jun 16;12(15):16295–309. doi: 10.1002/cam4.6242 (PMC10469732; doi:10.1002/cam4.6242)
Supplement: Supplementary file 1 — Table S1. [file CAM4-12-16295-s004.docx]

**Supplementary Table S1. Processing of different groups.**

|  | Groups | Co-culturewith 4T1 cell | Transfection | Co-culture with CD8+ T cell | |
| --- | --- | --- | --- | --- | --- |
| Section 3.2 | Control | - | - | - |  |
|  | TAMs | + | - | - |  |
|  | TAMs+si-NC | + | si-NC | - |  |
|  | TAMs+si-YAP | + | si-YAP | - |  |
| Section 3.3 | TAMs. | + | - | - |  |
|  | oe-NC | + | oe-NC | - |  |
|  | oe-YAP | + | oe-YAP | - |  |
|  | oe-YAP+si-NC | + | oe-YAP, si-NC | - |  |
|  | oe-YAP+si-STAT3 | + | oe-YAP, si-STAT3 | - |  |
| Section 3.4 | si-NC(1:3) | + | si-NC | + |  |
|  | si-NC(1:5) | + | si-NC | + |  |
|  | si-NC(1:10) | + | si-NC | + |  |
|  | si-YAP(1:3) | + | si-NC | + |  |
|  | si-YAP(1:5) | + | si-NC | + |  |
|  | si-YAP(1:10) | + | si-NC | + |  |
|  | si-YAP+NC-oe-STAT3 | + | si-YAP, NC-oe-STAT3 | + |  |
