# Supplementary figures and images for: YAP/STAT3 inhibited CD8 + T cells activity in the breast cancer immune microenvironment by inducing M2 polarization of tumor‐associated macrophages
Source: Cancer Med. 2023 Jun 16;12(15):16295–309. doi: 10.1002/cam4.6242 (PMC10469732; doi:10.1002/cam4.6242)

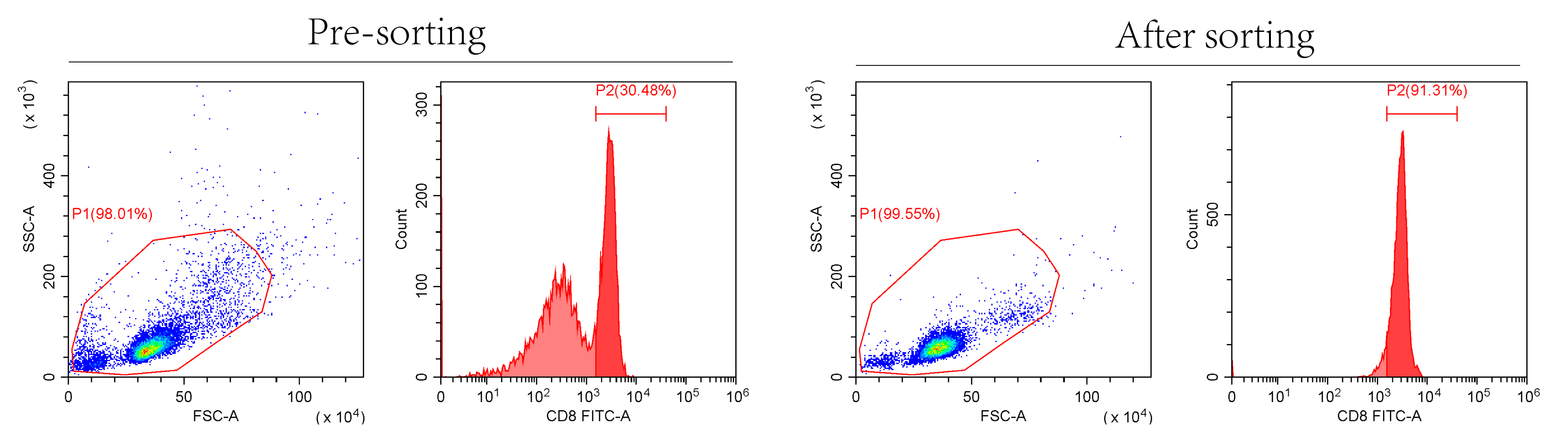

Supplement: Supplementary file 2 — Figure S1. [file CAM4-12-16295-s003.jpg]

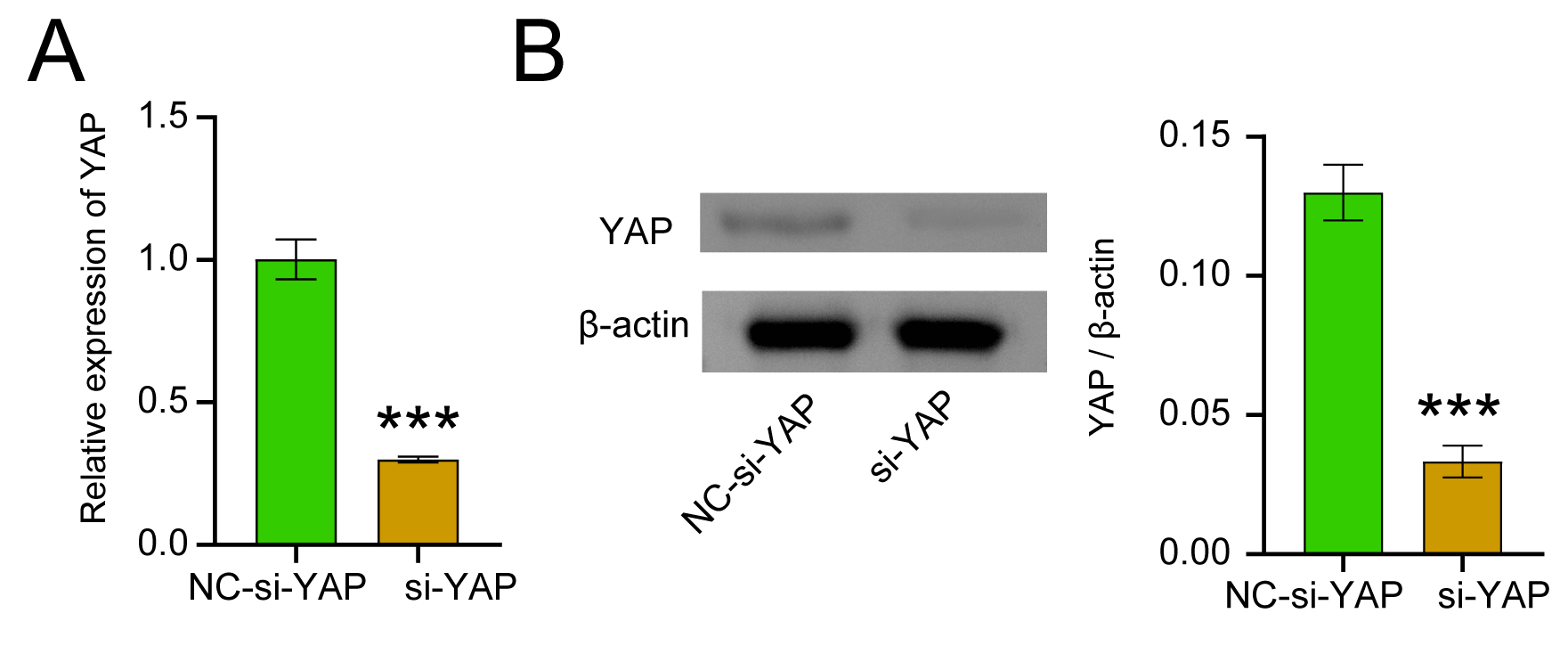

Supplement: Supplementary file 3 — Figure S2. [file CAM4-12-16295-s005.jpg]

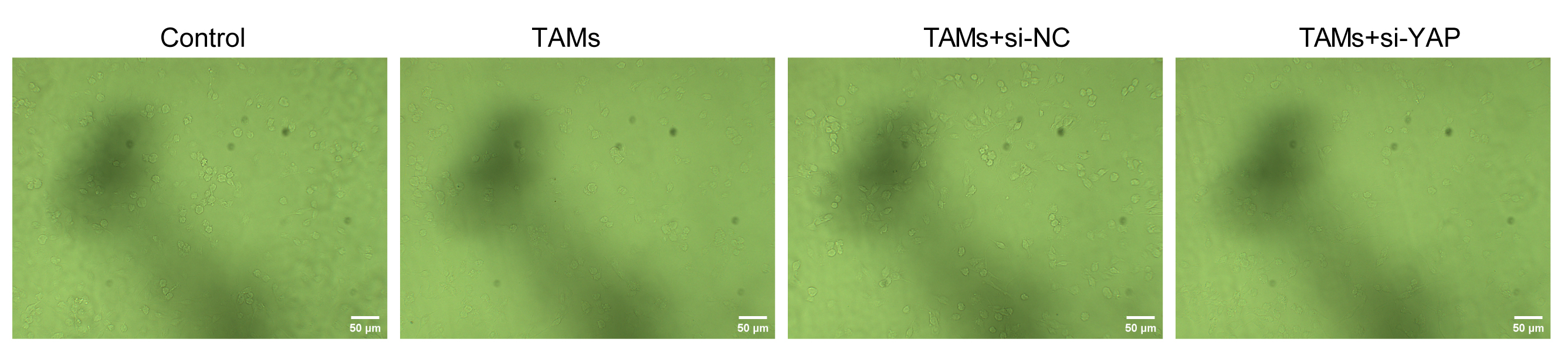

Supplement: Supplementary file 4 — Figure S3. [file CAM4-12-16295-s001.jpg]
