# Supplementary material for: YAP/STAT3 inhibited CD8 + T cells activity in the breast cancer immune microenvironment by inducing M2 polarization of tumor‐associated macrophages
Source: Cancer Med. 2023 Jun 16;12(15):16295–309. doi: 10.1002/cam4.6242 (PMC10469732; doi:10.1002/cam4.6242)
Supplement: Supplementary file 5 — Figures S1–S3 Captions [file CAM4-12-16295-s002.docx]

Figure S1. Isolated CD8^+^ T cells were identified by flow cytometry.

**Figure** **S2. Transfection efficiency verification experiment.** A. YAP levels were measured using WB. B. YAP levels were detected using RT-qPCR. ***P < 0.001, **P < 0.01, *P < 0.05 vs the si-NC group. t-test.

Figure S3. White light image of Figure 2E.
